# Cell wall proteomics of crops

**DOI:** 10.3389/fpls.2013.00017

**Published:** 2013-02-07

**Authors:** Setsuko Komatsu, Yuki Yanagawa

**Affiliations:** ^1^National Institute of Crop Science, National Agriculture and Food Research OrganizationTsukuba, Japan; ^2^Plant Science Center, RIKEN Yokohama InstituteYokohama, Japan

**Keywords:** crop, proteomics, cell wall, drought stress, flooding stress

## Abstract

Cell wall proteins play key roles in cell structure and metabolism, cell enlargement, signal transduction, responses to environmental stress, and many other physiological events. Agricultural crops are often used for investigating stress tolerance because cultivars with differing degrees of tolerance are available. Abiotic and biotic stress factors markedly influence the geographical distribution and yields of many crop species. Crop cell wall proteomics is of particular importance for improving crop productivity, particularly under unfavorable environmental conditions. To better understand the mechanisms underlying stress response in crops, cell wall proteomic analyses are being increasingly utilized. In this review, the methods of purification and purity assays of cell wall protein fractions from crops are described, and the results of protein identification using gel-based and gel-free proteomic techniques are presented. Furthermore, protein composition of the cell walls of rice, wheat, maize, and soybean are compared, and the role of cell wall proteins in crops under flooding and drought stress is discussed. This review will be useful for clarifying the role of the cell wall of crops in response to environmental stresses.

## Introduction

The cell wall is an important sub-cellular organelle for the modulation of some stress signals and an important target of stress response-related changes in protein abundance. In addition to sensing environmental stresses, plant cell walls are dynamic structures that are essential for cell division, enlargement, and differentiation (Roberts, 2001; Huckelhoven, 2007). Although cell wall proteins account for only 10% of the extracellular matrix mass, they comprise several hundred different molecules with diverse cellular functions, including cell structure maintenance and responses to abiotic and biotic stresses (Somerville et al., 2004). Furthermore, it has been demonstrated in yeast cells that transient damage to the cell wall induces cell wall-related genes as part of a homeostatic response to maintain cell integrity (Garcia et al., 2004). However, it remains to be elucidated if plant cells utilize a similar mechanism to that found in yeast. Thus, crop cell wall proteomics could provide further information of the underlying mechanisms of plant responses to environmental stresses, which may prove useful for the bioengineering of more tolerant crops.

The cell wall is also thought to an important material as a biomass material, because it consists of layers primarily composed of cellulose and hemicelluloses, which are polymers of β-1,4 linked glucose and mixtures of sugars such as xylose, mannose, and galactose, respectively, being sugar sources of ethanol fermentation. Bioethanol is produced by ethanol fermentation from sugars such as glucose, fructose, and galactose. Maize and sugarcane are being used to produce bioethanol due to their high productivity and high levels of carbohydrates providing simple sugars; however, these plants are also important crops to us as food supplies. To avoid the use of food crops, the identification of alternative source species for bioethanol production is desirable. The plant cell wall consists of cellulose, hemicellulose and pectins (Demura and Ye, 2010; Pauly and Keegstra, 2010) which, upon acid or enzymatic hydrolysis can yield monosaccharides for bioethanol production. The non-edible portions of plants, such as rice straw and sugarcane pomace, and other crops including cell wall components are potential sources of sugar needed for bioethanol production, although low productivity of bioethanol is a problem to be solved. To overcome this, these plants need improvements of cell wall components to provide high quality and a large quantity of sugar sources producing high productivity of bioethanol. Thus, cell wall proteomic studies are expected to provide useful information for understanding the mechanisms controlling quality and quantity of plant cell wall components. However, such a research is very little.

Various cell wall proteins have been characterized in *Arabidopsis* (Bayer et al., 2006; Minic et al., 2007; for review Jamet et al., 2006, 2008a,b; Zhang et al., 2011), *Medicago* (Watson et al., 2004; Soares et al., 2007), chickpea (Bhushan et al., 2006), maize (Zhu et al., 2006), rice (Jung et al., 2008; Chen et al., 2009; Cho et al., 2009), and potato (Lim et al., 2012). In addition, many types of stress-associated cell wall proteins have been identified in crops, including flooding stress-induced proteins in soybean (Komatsu et al., 2010) and wheat (Kong et al., 2009), drought stress-induced proteins in rice (Pandey et al., 2010), maize (Zhu et al., 2007), and chickpea (Bhushan et al., 2007), hydrogen peroxide-induced proteins in rice (Zhou et al., 2011), and/or pathogen-induced proteins in maize or tomato (Chivasa et al., 2005; Dahal et al., 2010). Also, cell wall proteins have been studied in wounded *Medicago* (Soares et al., 2009). Although many proteomic studies of primary cell wall have been conducted in *Arabidopsis* (Chivasa et al., 2002; Boudart et al., 2005; Jamet et al., 2006, 2008a), there have correspondingly fewer proteomic studies devoted to systematically mapping the proteins of the secondary cell wall (Millar et al., 2009). The utility of plant secondary cell wall biomass for industrial and biofuel purposes depends upon improving cellulose amount, availability, and extractability. The possibility of engineering such biomass requires much more knowledge of the genes and proteins involved in the synthesis, modification and assembly of cellulose, lignin and xylans (Millar et al., 2009).

Research on the plant cell wall has primarily focused on carbohydrate components due to their structural role and commercial value, whereas study of the complex mechanisms of stress responses mediated by cell wall proteins has remained secondary (Bhushan et al., 2007). In this review, the current methods of purification and purity test of crop cell wall proteins are presented, and the results of protein identification using gel-based and gel-free proteomic techniques are described. Furthermore, the role of cell wall proteomics of rice, wheat, maize and soybean under flooding and drought stresses is discussed.

## Cell wall purification and purity test

Cell wall proteins can be classified into three categories according to their interaction with other cell wall components (Jamet et al., 2006). The first is a soluble protein group, which has little or no interaction with cell wall components and thus moves freely in the extracellular space. Such proteins can be found in the culture media of cell suspensions and seedlings or can be extracted with low ionic strength buffers. The second is a group of weakly bound cell wall proteins that bind the extracellular matrix by Van der Waals forces, hydrogen bonds, and hydrophobic or ionic interactions. These proteins can be extracted from cell walls using salts. The third is a group of strongly bound cell wall proteins, and there is no efficient procedure to release these proteins from the extracellular matrix up to now. Within the past few years, there have been rapid advances in cell wall research (Jamet et al., 2008a).

The purification of plant cell walls is hampered by a number of technical difficulties such as contamination from other organelles. Thus, characterization of the cell wall proteome remains challenging and requires a combination of various treatment and analytical approaches (Watson et al., 2004). For example, mass spectrometry (MS) analyses have identified many proteins not previously believed to be extracellular, while multidimensional peptide analysis has facilitated the identification and characterization of over 250 *Arabidopsis* cell wall proteins, including new subsets of proteins (Bayer et al., 2006; Rossignol et al., 2006). With this approach, the presence of numerous extracellular proteases in the matrix was also confirmed (Bayer et al., 2006; Rossignol et al., 2006). For *Arabidopsis*, Jamet et al. (2008b) described a protocol for purifying soluble and weakly bound cell wall proteins with only low levels of contamination by intracellular proteins, thus providing a more acurate description of protein functions in the apoplast (Jamet et al., 2008b). However, extraction methods developed for *Arabidopsis* may not be useful for other species. Structural differences in the cell walls between species could have consequences to cell susceptibility to rupture by infiltration techniques, whereas compositional differences in the matrix, such as the content of homogalacturonic acid, might require the use of different extraction buffers for more effective protein release from the matrix. This implies that for the first proteomic studies of a plant species, the level of contamination of the cell wall extract must be monitored carefully and the extraction protocol adjusted accordingly to maximize its content of cell wall proteins. Here, the methods of purification and purity assay of cell wall proteome fraction from crops are explained.

### Rice

Rice production in Asia has doubled since 1961 due to the breeding of new rice cultivars using intensive cultivation systems. In addition to being an important agricultural crop, rice is a useful model plant for biological research because it has a smaller genome than those of other cereals (Devos and Gale, 2000). The International Rice Genome Sequencing Project (2005) produced a map-based, high-quality sequence covering 95% of the 389-Mb rice genome. The annotation of the rice genome has progressed rapidly (The Rice Annotation Project, 2007), and a high proportion of the predicted genes are supported by full-length cDNAs (Rice Full-Length cDNA Consortium, 2003). Since completing the rice genome sequence, the challenge for the rice research community has been to identify the function and regulation of rice genes and proteins. Proteomic studies, including those directed toward the cell wall proteome, are expected to improve our understanding of these processes.

Chen et al. (2009) presented a proteomic analysis of weakly bound rice cell wall proteins extracted from rice calli with mannitol/CaCl_2_, followed by back extraction with water-saturated phenol. The isolated cell wall proteins were evaluated for contamination with cytosolic proteins by measuring glucose-6-phosphate dehydrogenase activity, which indicated the presence of only low levels of intracellular proteins and a significant enrichment of cell wall proteins. Using multidimensional protein identification technology (MudPIT), a total of 292 proteins were identified, and bioinformatic analysis showed that 72.6% of these proteins possessed a signal peptide, indicating a total of 198 (67.8%) proteins were determined to be cell wall proteins in rice. Functional classification divided the extracellular proteins into different groups, including glycosyl hydrolases (23%), antioxidant proteins (12%), cell wall structure-related proteins (6%), metabolic pathways (9%), protein modification (4%), defense (4%), and protease inhibitors (3%). Furthermore, comparative analysis of the identified rice cell wall proteins with those of *Arabidopsis* identified 25 novel cell wall proteins that were specific to rice (Chen et al., 2009).

High-purity secreted rice proteins were obtained from the medium of a suspension of callus culture (Cho et al., 2009). To check for contamination from other cellular compartments, Western blot analyses were performed using antibodies specific for the cytoskeletal actin protein and Hsp70, and showed that the purified rice proteins were secretory. Using MudPIT, a total of 555 rice proteins were identified, and bioinformatics analysis indicated that 154 proteins (27.7%) were considered to be secreted proteins because they possessed a signal peptide. Functional classification divided the majority of identified proteins into stress response proteins (27%), metabolic proteins (26%) and factors involved in protein modification (24%). Comparative analysis demonstrated that one third of the secreted rice proteins overlapped with those of *Arabidopsis*. Furthermore, 25 novel rice-specific secreted proteins were found (Cho et al., 2009).

Using MudPIT, the two proteomic studies described above identified more than 150 secreted or cell wall proteins, which included 25 novel rice-specific secreted or cell wall proteins. Out of rice specific secreted and/or cell wall proteins, LYM1/LYM2 and gibberellin-regulated proteins were the same between secreted and/or cell wall proteins. Two proteins, LYM1 and LYM2, which contain LysM (lysin motif) domains, a widely distributed protein motif which binds to peptidoglycans and chitin, most likely recognize the N-acetylglucosamine moiety (Cho et al., 2009). In addition, two gibberellin-regulated proteins belonging to the GASA family were increased by gibberellin and might have functions in plant development as shown in expression studies during silique development and seed germination (Chen et al., 2009). These results indicate that MudPIT is a suitable technique for cell wall proteomics.

### Maize

Maize is an important food crop as well as a classic genetic model plant, and has been used to explore the genetic mechanism of heterosis because it exhibits high levels of phenotypic (Flint-Garcia et al., 2005), sequence (Schnable et al., 2009), transcriptional (Ma et al., 2006; Stupar and Springer, 2006), and translational variation (Hoecker et al., 2008).

Zhu et al. (2006) performed the cell wall proteomic analysis of maize. In the paper, they presented the effectiveness of a vacuum infiltration–centrifugation technique for extracting water-soluble and loosely ionically bound cell wall proteins from the root elongation zone of maize. The purity of the extracted cell wall proteins was evaluated by comparison with total soluble proteins extracted from homogenized tissue. In addition, MS analysis of proteins separated on two-dimensional gel electrophoresis (2-DE) indicated that 84% of the cell wall proteins differed from the total soluble proteins. It means that only about 16% of the proteins identified overlapped with those from the total soluble protein gel. Several lines of evidence indicated that the vacuum infiltration-centrifugation technique effectively enriched for cell wall proteins. Approximately 40% of the loosely ionically bound cell wall proteins had traditional signal peptides and 33% were predicted to be non-classical secretory proteins, whereas only 3 and 11%, respectively, of the total soluble proteins were classified into these categories. Many of the identified cell wall proteins were previously shown to be involved in cell wall metabolism and cell elongation. Primary cell walls can be divided into two types: type I cell wall were found in all flower plants expect the grass family and type II cell wall were in the grass family. The identified cell wall proteins of maize were type II cell walls, were not detected in proteomic studies that focused on type I cell walls. These newly identified proteins included endo-1,3;1,4-β-D-glucanase and α-L-arabinofuranosidase, which act on the major polysaccharides of type II cell walls (Zhu et al., 2006). Furthermore, Malate dehydrogenase and β-glucosidase, which were found in apoplastic fluid in barley and oat primary leaves, were identified in maize. Enolase, which was detected in the cell walls of *Candida albicans*, *Arabidopsis*, and *Medicago sativa*, was also identified in maize (Zhu et al., 2006). With this approach, several novel cell wall proteins of maize were found. The same purification technique was also used for the identification of cell wall proteins involved in drought stress (Zhu et al., 2007).

### Chickpea

Legumes are valuable agricultural and commercial crops that serve as important nutrient sources for the human diet and animal feed. Legume plants typically form symbiotic relationships with both nitrogen-fixing bacteria and arbuscular mycorrhizal fungi. Many secondary metabolites in legumes have been implicated in defense against pathogen and are of particular interest as novel pharmaceuticals (Haridas et al., 2001). Legume such as soybean is one group of the most important agricultural crops in many countries. As the entire genome sequences of numerous legumes have been completed (Sato et al., 2008; Schmutz et al., 2010; Young et al., 2011; Katayose et al., 2012), proteomic approaches to identify and quantify protein molecules have been facilitated.

To better understand the function of the extracellular proteins in chickpea, Bhushan et al. (2006) examined its' extracellular proteome. In their study, chickpea seedling powder was added to homogenizing buffer (Averyhart-Fullard et al., 1988), and the cell wall fraction was recovered by differential centrifugation. Microscopy showed that the extracellular matrix fraction was free of plasma membranes and other ultrastructural cytoplasmic organelles. This finding was confirmed by performing catalase and ATPase activity assays to assess contamination by cytosol components and plasma membrane, respectively. Using 2-DE and MALDI-TOF-MS together with LC-MS/MS, 131 spots were analyzed, of which a total of 121 proteins were detected. Functions were assigned to 69 extracellular matrix proteins, whereas 43 proteins belonged to unidentified function categories, and 9 had no significant match. The proteins were classified into six different functional classes, which included metabolism (19%), cell signaling (13%), cellular transport (10%), development (9%), stress response (6%), and unidentified function.

Bhushan et al. (2006) also reported evidence for the presence of several unexpected proteins in the cell wall fraction with known biochemical activities that had never been associated with the extracellular matrix. Although many researchers have reported non-canonical proteins in cell wall fractions, this is not sufficient to unambiguously verify that the extracellular matrix fraction in this study is without any contamination as suggested by the presence of RuBisCO subunits (Wang et al., 2004). The presence of RuBisCO is most likely inevitable because it is the most abundant plant protein and has been shown in several earlier reports (Wang et al., 2004; Watson et al., 2004; Bhushan et al., 2006). This purification technique was further used for the identification of cell wall proteins of chickpea involved in drought stress (Bhushan et al., 2007).

## Cell wall proteomics under stresses

Climate change poses tremendous global challenges for agriculture, as plant growth and productivity are strongly influenced by environmental stresses. As the global climate changes, high temperature, flooding, and drought are becoming the most important environmental factors influencing the yield and quality of crops. Research conducted to date on changes in the cell wall proteome in response to environmental stress is summarized here (Table 1). Despite significant efforts, quite a large number of cell wall proteins remain unidentified up to date. To investigate the function of cell wall proteins under abiotic and biotic stresses, the remaining unidentified proteins should be identified.

**Table 1:** **Summary of published cell wall proteomics analyses in response to environmental stress**.

**Plant**	**Stress**	**Proteomics techniques**	**IP^a^**	**Major findings**	**References**
Rice	Dehydration	2-DE	94	Dehydration-responsive proteins mainly involved in a variety of functions, including carbohydrate metabolism, cell defense and rescue, cell wall modification, cell signaling and molecular chaperones	Pandey et al., 2010
		LC-TOF MS			
Rice	Hydrogen peroxide	2-DE	54	These proteins identified are involved in a variety of processes, including redox homeostasis, cell wall modification, signal transduction, cell defense and carbohydrate metabolism, indicating a complex regulative network in the apoplast of seedling roots under H_2_O_2_ stress	Zhou et al., 2011
		MALDI TOF/TOF MS			
Maize	Water deficit	2-DE	152	Water stress-responsive proteins were identified and categorized, into five groups; apoplastic ROS level increases in the apical region of the elongation zone of water stressed maize roots and hence provides novel information about the complex mechanisms regulating root growth under water stress	Zhu et al., 2007
		Q-TOF MS			
Maize	Pathogen	2-DE	12	Secretion of a new class of putative enzyme inhibitor, the apparent recruitment of classical cytosolic proteins into the cell wall, and the change in phosphorylation status of extracellular matrix proteins	Chivasa et al., 2005
		MALDI-TOF MS			
Chickpea	Dehydration	2-DE	134	More than hundred extracellular matrix proteins with a variety of cellular functions which are cell wall modification, signal transduction, metabolism, and cell defense and rescue, play crucial roles in dehydration stress sensing and tolerance mechanism	Bhushan et al., 2007
		LC-TOF MS			
Soybean	Flooding	2-DE	16	Two lipoxygenases, germin-like protein precursors, glycoprotein precursors, super oxide dismutase were decreased, and then lignification was suppressed under flooding stress	Komatsu et al., 2010
		MALDI-TOF MS and nanoLC MS/MS			
Wheat	Flooding	2-DE nanoLC MS/MS	18	Four proteins were upregulated and 14 proteins were down-regulated by flooding stress. Both gel-based proteomic system and LC-MS/MS-based proteomic system under flooding stress, most were disease/defense proteins	Kong et al., 2009
		Gel-free nanoLC MS/MS	15		

a IP, Number of identified proteins.

### Rice cell wall proteomics under drought stress and in response to hydrogen peroxide

For a better understanding of the underlying molecular mechanism of the dehydration stress response in rice seedlings, Pandey et al. (2010) used a cell wall proteomic technique. In this study, 4-week-old rice seedlings were subjected to progressive dehydration by withdrawing water, and proteomic analyses of the drought-induced changes in the extracellular matrix proteins were performed using 2-DE. Dehydration-responsive temporal changes in protein levels indicated that 192 proteins had individual intensities that differed by more than 2.5-fold from baseline levels at one or more time points during the dehydration stress. The proteomic analysis led to the identification of nearly 100 differentially regulated proteins that were predicted to be involved in a variety of cellular functions including carbohydrate metabolism, cell defense and rescue, cell wall modification, cell signaling, protein folding, and stabilization, and all of which were suggested to play key roles in the dehydration tolerance cascade (Pandey et al., 2010).

The plant apoplast is a major site for signal transduction and plays a major role in defensive responses to environmental stresses. Zhou et al. (2011) reported their comparative proteomic analysis of the root apoplasts of rice seedlings in response to hydrogen peroxide. Two week-old rice seedlings were treated with low concentrations of hydrogen peroxide and a modified vacuum infiltration method was used to extract apoplastic proteins from the roots. A 2-DE analysis revealed 58 differentially expressed protein spots under low hydrogen peroxide conditions. Of these, 54 protein spots were identified by MS as matches to 35 different proteins including known and novel hydrogen peroxide-responsive proteins. Almost all of these identities (98%) were indeed apoplast proteins confirmed either by previous experiments or through publicly available prediction programs. These identified proteins were involved in a variety of processes, including redox homeostasis, cell wall modification, signal transduction, cell defense, and carbohydrate metabolism, indicating a complex regulative network in the apoplast of seedling roots under hydrogen peroxide stress (Zhou et al., 2011). They indicated that the identified proteins might work cooperatively to establish a complex network of apoplast response to exogenous hydrogen peroxide in the rice seedling root, and depict the strategies of the root apoplast to oxidative challenge.

### Maize cell wall proteomics under drought stress and in response to pathogens

To gain a comprehensive understanding of how cell wall protein composition changes in association with the differential growth responses to water deficit in different regions of the elongation zone, Zhu et al. (2007) used a proteomic approach for water soluble and loosely ionically bound cell wall proteins. As different regions, 20 mm of each root was sectioned into four regions (distances are from the root cap junction): R1, 0–3 mm plus the root cap; R2, 3–7 mm; R3, 7–12 mm; R4, 12–20 mm. The analyses demonstrated region-specific changes in the protein profiles of control and water-stressed roots. In total, 152 water stress-responsive proteins were identified and categorized into five functional groups: metabolism of cell wall reactive oxygen species, defense and detoxification, hydrolases, carbohydrate metabolism and other/unknown functions. In particular, the changes in protein abundance related to reactive oxygen species metabolism predicted an increase in apoplastic reactive oxygen species production in the apical region of the elongation zone of water-stressed roots. This was verified by quantification of hydrogen peroxide content in extracted apoplastic fluid and by *in situ* imaging of apoplastic reactive oxygen species levels. This response could contribute directly to the enhancement of wall loosening in this region (Zhu et al., 2007). This large-scale proteomic analysis provided novel insights into the complexity of mechanisms that regulated root growth under water deficit conditions and highlighted the spatial differences in cell wall protein composition in the root elongation zone.

Chivasa et al. (2002) have reported their proteomic analysis of elicited maize suspension cultures. Using phosphotyrosine antibodies raised against synthetic phosphotyrosine peptides, they identified a number of phosphotyrosine protein spots secreted into the growth medium of cell cultures. Elicitor treatment of cell cultures-induced a rapid change in the phosphorylation status of extracellular peroxidases, the apparent disappearance of a putative extracellular β-*N*-acetylglucosamonidase, and accumulation of a putative secreted xylanase inhibitor protein. The onset of the defense response was accompanied by accumulation of glyceraldehyde-3-phosphate dehydrogenase and a fragment of a putative heat shock protein. Several distinct spots of both proteins, which preferentially accumulated in cell wall protein fractions, were identified. The novel observations of the secretion of a new class of putative enzyme inhibitor, the apparent recruitment of classical cytosolic proteins into the cell wall, and the change in phosphorylation status of extracellular matrix proteins, suggested that the extracellular matrix plays a complex role in defense (Chivasa et al., 2002). Taken together, it is suggested that cell wall proteins may have an important role in stress signal transduction.

### Chickpea cell wall proteomics under drought stress

Comprehensive extracellular matrix proteins analysis of chickpea under dehydration stress has been performed using comparative proteomics. This approach led to the identification of 134 differentially accumulated proteins, which include both predicted and novel dehydration-responsive proteins. This comparative proteomic study demonstrates that more than 100 extracellular matrix proteins with a variety of cellular functions, such as cell wall modification, signal transduction, metabolism, and cell defense and rescue, play crucial roles in dehydration stress sensing and tolerance mechanisms in chickpea (Bhushan et al., 2007).

### Soybean cell wall proteomics under flooding stress

To investigate the cell wall function of soybean under flooding stress, cell wall proteomics was performed using cell wall proteins purified from 2-day-old soybean plants treated with flooding for 2 days. The purity of the cell wall protein extract was confirmed by measuring the activity of glucose-6-phosphate dehydrogenase, a cytoplasmic marker enzyme. Using 2-DE and MS, it was found that 16 out of 204 cell wall proteins responded to flooding stress. Of these, two lipoxygenases, four germin-like protein precursors, three stem glycoprotein precursors and one Cu-Zn superoxide dismutase were found to be present at lower levels than those found in the control plants. These changed proteins indicated that the roots and hypocotyls of soybean under flooding stress suppressed lignification through decreasing these cell wall proteins by down-regulation of reactive oxygen species and inhibition of jasmonate biosynthesis (Komatsu et al., 2010).

### Wheat cell wall proteomics under flooding stress

A procedure for extracting and purifying cell wall proteins was adopted for wheat seedling roots, and the purity of the cell wall protein extract was confirmed by measuring the activity of glucose-6-phosphate dehydrogenase. To identify flooding-stress responsive proteins in the wheat cell wall, gel-based and LC-MS/MS-based proteomic techniques were applied. A total of 18 and 15 proteins were shown to accumulate in response to flooding by the former and latter proteomic techniques, respectively. Among the proteins detected at lower levels in response to flooding, most were related to the glycolysis pathway and cell wall structure and modification. In contrast, the cell wall proteins of highest abundance after flooding treatment belonged to the category of defense and disease-response proteins. Among the identified proteins, only methionine synthase, β-1,3-glucanases and β-glucosidase were consistently detected by both techniques. The decrease of these three proteins suggested that wheat seedlings respond to flooding stress by restricting cell growth to avoid energy consumption; by coordinating methionine assimilation and cell wall hydrolysis, cell wall proteins played critical roles in flooding responsiveness (Kong et al., 2009).

## Differences in stress responses among crops

### Drought stress

Water deficit and dehydration are the most crucial environmental factors that limit the productivity and geographic distribution of many crop species. Evidence suggests that dehydration-responsive changes in protein levels may lead to cellular adaptation against water-deficit conditions. Plants exposed to dehydration mostly rely on the protection of cellular integrity to prevent mechanical damage by changing the composition of the cell wall (Vicre et al., 2004; Moore et al., 2006).

Using cell wall proteomic techniques, the mechanisms of drought response in the cereals rice (Pandey et al., 2010) and maize (Zhu et al., 2007), and in the legume chickpea (Bhushan et al., 2007) were analyzed. In the case of chickpea and rice, cell wall proteins were purified from shoots, whereas root tissue was used for the analysis of maize. Although many proteins unique to each crop were identified, proteins associated with cell defense and rescue, ascorbate peroxidases and glyoxalase, respectively, and one protein involved in carbohydrate metabolism, fructose-bisphosphate aldolase, were common in all three crops. It has been proposed that functionally important proteins are more evolutionarily conserved than less vital proteins.

Pandey et al. (2010) speculated that the preponderance of differential protein networks among crop species may be attributable to the evolutionary species-specific dynamics of the cell wall proteomics because the protein expression profile is a reflection of the cellular environment and ecological niche of the corresponding organism. A closer look at their results indicated that chickpea and rice share many common proteins in shoot. Notably, methyltransferases and the chaperone class of proteins were absent in maize exposed to drought stress, suggesting the type of sampled tissue can influence the proteomics. Interestingly, rice and maize showed the highest degree of commonality, possibly because both species belong to the Poaceae family. Together, these data provide evidence for molecular diversity among different plant species as opposed to commonality with respect to protein profiles at the level of the organism and/or tissue. The higher percentage of crop-specific dehydration-responsive proteins identified in this study signifies that examining the cell wall proteomics of different crops, particularly the major lineages of higher plants, at different tissue levels is needed to better understand the critical role of the cell wall in drought stress tolerance (Pandey et al., 2010).

### Flooding stress

Flooding caused by heavy or continuous rainfall in an area with poorly drained soil can be a devastating environmental stressor, as many types of crops cannot tolerate flooding. Soybean and other crops are particularly susceptible to stress arising from flooding (Komatsu et al., 2012). Previous studies showed that genes encoding transcription factors (Liu et al., 2005), signal transduction components (Baxter-Burrell et al., 2002), non-symbiotic hemoglobin (Dordas et al., 2004), ethylene signaling components (Reggiani, 2006), as well as genes involved in nitrogen metabolism (Mattana et al., 1994) and cell wall loosening (Saab and Sachs, 1996), were up-regulated under low-oxygen condition. Since flooding produces hypoxia in plant tissues, these genes may be regulated by flooding.

The primary cell wall plays in regulating extension growth, cell adhesion, and cell morphology (Cosgrove, 2001). Pereira et al. (2011) reported that the primary cell wall is essentially a hydrated matrix and sensitive to global changes in the water content of the plant by altering its biophysical properties. The partial adaptation to flooding stress might be required a compensatory modification of the extracellular matrix. The mechanisms underlying the flooding response in soybean (Komatsu et al., 2010) and wheat (Kong et al., 2009) have been analyzed using cell wall proteomic techniques. Although soybean is a dicot and wheat is a monocot, the roots of both crops were affected by flooding stress. In fact, the physiological and morphological response to flooding stress was to suppress root growth (Kong et al., 2009; Komatsu et al., 2012). In soybean and wheat, the levels of methionine synthase were commonly reduced under flooding stress, indicating that both crops restrict cell growth to avoid energy consumption by coordinating methionine assimilation and cell wall hydrolysis, which is consistent with the observed growth suppression of wheat seedlings by flooding stress. However, many kinds of proteins differentially accumulated in soybean and wheat under flooding stress. In roots and hypocotyls of soybean, lignification was suppressed by the down regulation of reactive oxygen species and jasmonate biosynthesis. On the other hands, in the case of wheat, the reduction of cell wall hydrolytic enzymes in response to flooding stress might allow not only cell wall polysaccharides to be used as carbohydrate sources, but also restricts cell elongation, thus better preparing wheat seedlings for long periods of submergence.

## Future perspective

Plant cell walls are involved in both maintenance of the cell structure and resistance against environmental stresses, because the location of the cell wall places it in direct contact with environmental factors. Indeed, flooding stress leads to morphological suppression of roots, and in severe cases, the root tissue is destroyed, as was demonstrated in soybean (Kong et al., 2009; Komatsu et al., 2012). When plant pathogens and insects attack plants, the cell wall represents the first line of host defense and resistant to damage. Thus, examination of the structural and functional mechanisms underlying stress response in the cell wall is expected to aid in the development of stress-tolerant crops. Based on the data of proteomic analysis of cell wall, the future of abiotic stress research should focus on targeting multiple gene regulation, because the change on a single gene expression may not have enough results on the field in question, since genetic regulation is complex. It may be possible to generate stress-tolerant crops using mutants with loss/gain of function in cell wall stress signal transduction mechanisms. Furthermore, the identified factors may aid in the genetic screening for crop tolerance against other stresses. It could be that proteomics is a useful tool to identify targets/markers proteins possibly involved in stress response of crops and to compare proteins identified in rice, wheat, maize, and soybean upon flooding and drought stresses.

### Conflict of interest statement

The authors declare that the research was conducted in the absence of any commercial or financial relationships that could be construed as a potential conflict of interest.

## Abbreviations

MudPIT: multidimensional protein identification technology.
